# Flexible coherent control of plasmonic spin-Hall effect

**DOI:** 10.1038/ncomms9360

**Published:** 2015-09-29

**Authors:** Shiyi Xiao, Fan Zhong, Hui Liu, Shining Zhu, Jensen Li

**Affiliations:** 1 School of Physics and Astronomy, University of Birmingham, Birmingham B15 2TT, UK; 2 National Laboratory of Solid State Microstructures & School of Physics, Collaborative Innovation Center of Advanced Microstructures, Nanjing University, Nanjing 210093, China

## Abstract

The surface plasmon polariton is an emerging candidate for miniaturizing optoelectronic circuits. Recent demonstrations of polarization-dependent splitting using metasurfaces, including focal-spot shifting and unidirectional propagation, allow us to exploit the spin degree of freedom in plasmonics. However, further progress has been hampered by the inability to generate more complicated and independent surface plasmon profiles for two incident spins, which work coherently together for more flexible and tunable functionalities. Here by matching the geometric phases of the nano-slots on silver to specific superimpositions of the inward and outward surface plasmon profiles for the two spins, arbitrary spin-dependent orbitals can be generated in a slot-free region. Furthermore, motion pictures with a series of picture frames can be assembled and played by varying the linear polarization angle of incident light. This spin-enabled control of orbitals is potentially useful for tip-free near-field scanning microscopy, holographic data storage, tunable plasmonic tweezers, and integrated optical components.

## Abstract (web summary)

Conventional methods to control surface plasmon polaritons with light offer limited tunability or complex design parameters. Here, Xiao *et al*. demonstrate coherent and independent control of surface plasmon polariton orbitals for two opposite spins using multiple rings of nano-slots on a metasurface

## Graphical abstract

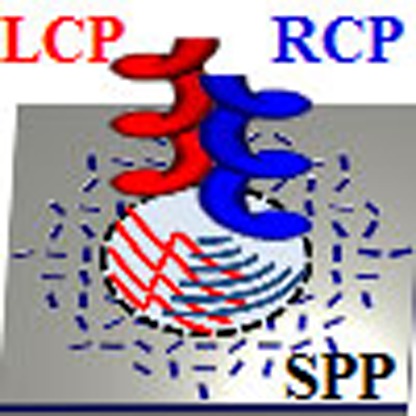

The subwavelength confinement of surface plasmon polariton (SPP) has been revolutionizing the way we control light at the nanoscale and holds promise for future optical information technology and optoelectronics^1^,^2^,^3^. Coupling propagating light to SPPs is therefore a primary interest for both fundamental studies and practical on-chip applications^4^. Prisms, holographic gratings and tailor-made plasmonic particles are commonly used for this purpose to compensate the momentum mismatch between SPPs and propagation waves^5^,^6^,^7^,^8^,^9^. However, these conventional methods usually provide a limited dynamic tunability unless sophisticated electrical or optical tuning on the material and geometrical parameters is employed^9^,^10^,^11^,^12^,^13^. On the other hand, many attentions have been paid to the spin–orbit interaction of light using geometric-phase-enabled optical and plasmonic systems^14^,^15^,^16^,^17^,^18^,^19^,^20^,^21^. Together with the recent developments of resonator-based^22–27^ and geometric-phase-enabled metasurfaces^28^,^29^,^30^,^31^,^32^,^33^, it offers an alternative route to excite SPPs through spin–orbit interaction with opposite geometric phases for the two spins. The associated spin-dependent phenomena can be regarded as the optical spin-Hall effect (OSHE)^34^,^35^,^36^,^37^,^38^,^39^,^40^ in a more general context about spin splitting of orbitals^18^,^32^,^33^,^41^,^42^,^43^. For example, a flip of the incident spin (circular polarization) can cause a split of beam displacement^18^,^42^, or a reverse in propagation direction^32^,^33^ of a propagating SPP. However, the time-reversely related SPP profiles, from the opposite geometric phases, generated with the two normal incident spins in these cases have so far only demonstrated simple and symmetric splitting of the two spins, known as OSHE. Without a proper geometric phase design scheme, the generated SPP profiles from OSHE are far from arbitrary and independent for the two spins. It refrains us to fully exploit the potential of OSHE and to allow the two spins to work cooperatively in a flexible manner.

Here we demonstrate coherent and independent control of SPP orbitals for the two opposite spins using multiple rings of nano-slots with properly designed orientations on a metasurface. These controls range from generating different focal spots to generating independent complicated profiles for the two spins. This is made possible in this work by establishing a geometric phase matching scheme, which gives the orientation profile of the nano-slots from the superimposition of the target SPP profiles for the two spins. This scheme provides us to achieve arbitrary OSHE. Resulting from this independence of spin splitting, we further demonstrate the two opposite spins can cooperate with each other. For example, we can dynamically tune the phases and amplitudes of the designated SPP orbitals. Such coherent control can further provide us the capability to assemble a series of individually designed ‘time' frames as a motion picture being played back by rotating the linear polarization of the incident light. This is a form of spin-enabled coherent control^44^,^45^,^46^ and provides a unique way in achieving tunable orbital motions in plasmonics. For example, it can be used as tip-free near-field scanning optical microscopy^47^, polarization-steering plasmonic tweezers^48^, and coherent inputs of SPP devices (coherent logic gate, transistor, etc.)^49^.

## Results

### Geometric phase design scheme

**Figure 1:**
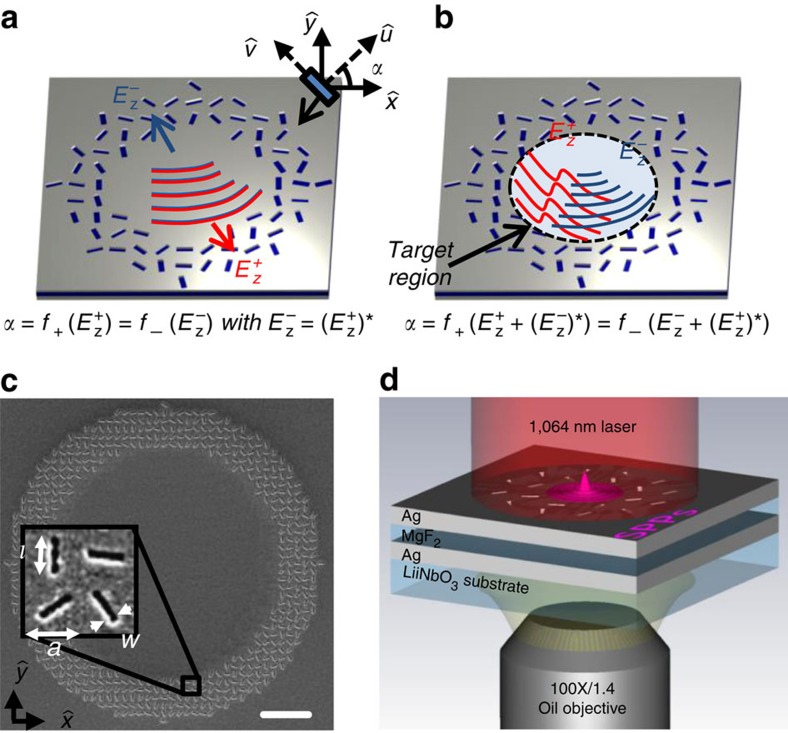
Arbitrary OSHE of SPP. (**a**,**b**) Identical nano-slots with orientation profile *α*(*x*,*y*) on the *x*−*y* plane with û, 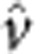 as the local principal axes of each particle. Light is incident normally on the surface. *α* can be designed by (**a**) direct substitution of the target SPP orbital *E*_z_^+^/*E*_z_^−^ (for LCP and RCP incident wave) into equation (1) with restriction 
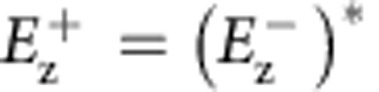
; or (**b**) the modified matching rule equation (2) employed in this work to gain independent control of local SPP orbitals generated within the ring of nano-slots. (**c**) Top-view scanning electron microscopy (SEM) image of experiment sample. The sample is with geometrical parameters defined as *l*=500 nm, *w*=50 nm and *a*=706 nm. Scale bars, 4 μm in (**c**). (**d**) Schematics of the experimental set-up.

Figure 1 shows our metasurface platform. It consists of two silver films separated by a dielectric spacer. An array of nano-slots on the upper film is etched with specific orientation profile *α*(*x*,*y*) (inset of Fig. 1a). A semiconductor laser at 1,064 nm is normally shined on the metasurface to generate a target SPP profile on the air-metal interface. For a left/right-handed circular polarization (LCP/RCP) incidence, each nano-slot reradiates as an electric dipole carrying an additional geometric phase ±2*α* (refs 27, 28, 29, 30, 31, 32, 33) in its cross-polarization radiation. By requiring constructive interference in building up a target SPP profile (see the derivation in Supplementary Note 1), we obtain the following function to design the *α* profile:


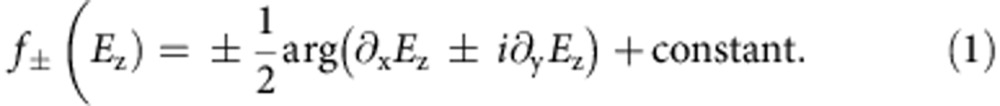


For a target SPP profile 
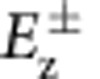
, we can use *α*(*x*,*y*)=*f*_+_
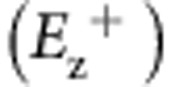
 for LCP incidence or equivalently *α*(*x*,*y*)=*f*_−_
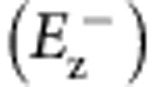
 for RCP incidence. This immediately imposes a usual restriction on the input target SPP orbital of the two spins: *E*_z_^+^=
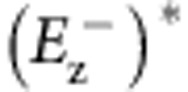
 for the thin layer of plasmonic particles to generate the same set of orientation profile. This time-reversal relationship actually comes from the opposite signs of the geometric phases for the two spins (the geometric origin of OSHE). Therefore, the two orbital profiles of different incident spins cannot be specified independently. We call it the direct scheme (Fig. 1a).

On the other hand, if we are only interested in generating SPP profiles within a slot-free region (the target region inside the ring of particles in Fig. 1), an arbitrary SPP profile without radiation into this region can be added to the input argument of equation (1). The orientation profile becomes different but still generates the same SPP profile. For a particular spin, if we decompose the SPP profile in the target region into inward and outward radiating parts. For example, a point-like standing wave 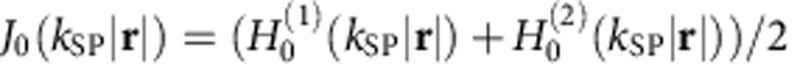, we only need to ensure the inward radiating part matches to the corresponding inward radiating part of the input argument *E*_z_ to equation (1). In other words, the information capacity carried by the orientation profile of the nano-slots is far from completely exhausted. This redundant information capacity by considering generating SPP in the target region can then be used for generating another SPP profile for the opposite spin. On the basis of this observation, we use a modified geometric phase scheme to obtain the orientation profile of the nano-slots:


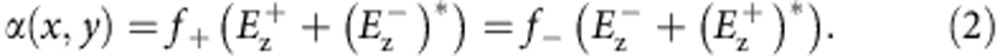


In the modified scheme, 
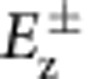
 is defined as the inward radiating part of the target profile for the particular spin while its conjugate version radiates in the outward direction. Therefore, the inward radiating parts of both input arguments to the function *f*_+_ and *f*_−_ become the expected 
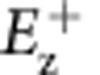
 and 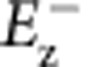 and both functions generate the same set of orientation profile of the nano-slots as required. Thus, we can make the spin-splitting arbitrarily specified. As we shall see, the scheme also allows arbitrarily complicated orbitals to be constructed, as long as we have enough number of nano-slots. The modified geometric phase matching scheme is schematically shown in Fig. 1b and is summarized in Supplementary Note 2. Here the key to achieve independent specification of the target SPP profiles of the two spins (
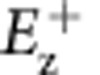
 and 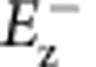) is to establish an appropriate geometric phase matching scheme. Our method is to superimpose the two target SPP profiles as a total input argument as in equation (2). By using the same set of atoms (without multiplexing) in generating SPP profiles for both LCP and RCP incidence, we have fully used the data capacity carried by the nano-slots, effectively a onefold increase compared with the direct scheme without using the superimposition. The same technique can also be readily applied in other applications like hologram generation^50^ so that the information capacity of the hologram can be doubled in storing two different holograms with real images for both LCP and RCP incidence using the same set of atoms, which is important for data storage application.

We note that the geometric phase, ±2*α*, at each particle is matched to 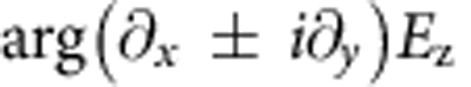 instead of simply arg(*E*_z_). We usually only use the scalar phase arg(*E*_z_) for phase matching without considering the vectorial nature of the propagation wave, for example, in generating unidirectional SPP propagation using geometric phase element or in holographic grating^6^,^7^,^8^,^9^,^31^,^32^,^33^. However, for two-dimensional (2D) SPP profiles (without a definite propagation direction) more generic than beam shaping^51^, for example, in tight focusing in the Fresnel regime (near-field) instead of the far-field regime, we have to consider also the additional phase contribution from ∂_*x*_±*i*∂_*y*_ (dependent on the local propagation direction of SPP, or equivalent the polarization direction on plane) if a precise control of profiles is needed. We note that the cross-polarization term radiates isotropically so that we do not need to consider the original fact that each slot should have an angle-dependent radiation efficiency. On the other hand, there is also a co-polarization radiation from each slot. These radiated fields at different dipoles are having the same phase, so that they do not interfere constructively. They can usually be neglected without interrupting the target SPP profiles, and can be further reduced by properly designing the plasmonic particles (see Supplementary Note 3 and Supplementary Figs 1 and 2). It is also worth to mention that the geometric phase matching rule equation (1) or equation (2) can be alternatively treated as a polarization-enabled holographic principle except that the plasmonic particles can now be placed at any locations (not every 2*π* phase change in conventional holograms) due to the flexible geometric phase added to the incident wave. The holographic-like scheme also means that it only ideally works for a single wavelength. Although our scheme does not provide dedicate dispersion compensation^52^, our design can tolerate a wavelength shift about ±5% with an acceptable performance as illustrated in Supplementary Fig. 3. Such bandwidth is wide enough for experiments and applications.

### Arbitrary and spin-dependent SPP profiles

**Figure 2:**
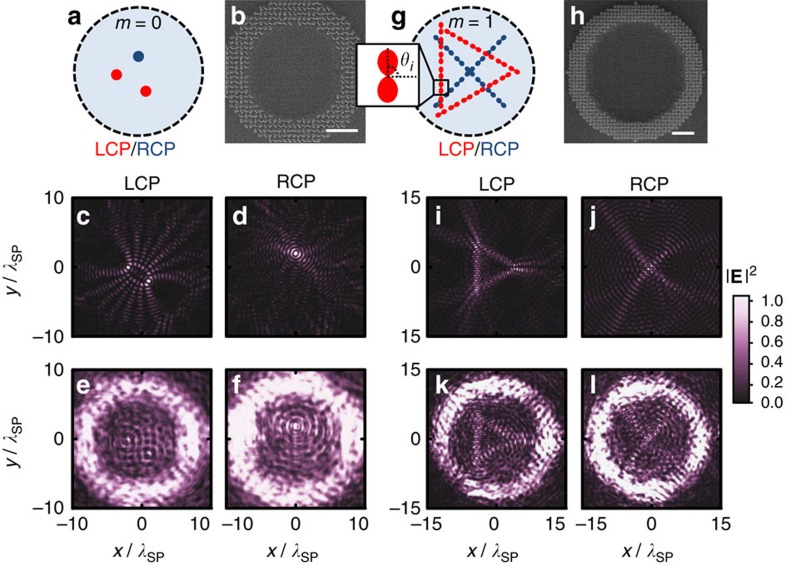
Arbitrary spin-Hall effect. (**a**) Arbitrary spin splitting: LCP orbital to focus to two spots (red) while RCP orbital to focus a single spot (blue) (see text for detailed specification). (**b**) Top-view SEM image of the fabricated sample: a ring of nano-slots (with radii from 6.6*λ*_SP_ to 10*λ*_SP_, embedded with a square array of particles separated by *a*=2*λ*_SP_/3). (**c**–**f**) are simulated 
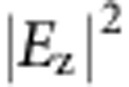
 and experimental intensity profiles for LCP and RCP incidence. (**g**) Arbitrary spin-dependent SPP profiles with more complicated pattern: shining LCP generating a triangle pattern (red) and shining RCP generating a cross pattern (blue). (**h**) Top-view SEM image of the fabricated sample (with radii from 10*λ*_SP_ to 13.3 *λ*_SP_ and *a*=2*λ*_SP_/3). (**i**–**l**) simulated 
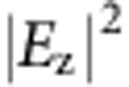
 and experimental intensity profiles for LCP and RCP incidence. Here we use same colour scales for different incident CP. Scale bars, 4 μm in **b**,**h** respectively.

As the first example, we demonstrate that the spin splitting of the generated orbitals of the two incident spins can be made completely arbitrary. We instruct the target orbitals for LCP incidence to consist of two focal spots at **r**_1_=(1, −2)*λ*_SP_, **r**_2_=(−2,0)*λ*_SP_ and the target orbital for RCP incidence as a single focal spot at **r**_3_=(0,2)*λ*_SP_ (schematically shown in Fig. 2a) where *λ*_SP_≈1.05 μm is the surface wave wavelength at the air/metal interface, that is, we set 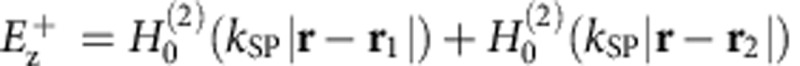, and 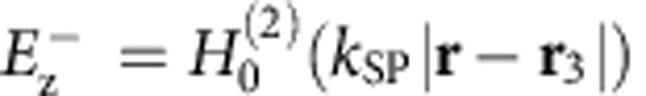, where *k*_SP_ is the in-plane wavenumber of SPP and 
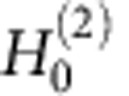
 is the zeroth-order second-kind Hankel function with inward radiation. A square array of nano-slots in a ring shape is then designed and fabricated with orientation profile by inserting 
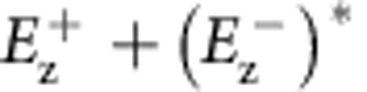
 to equation (2)(See designing recipe in Supplementary Note 2). Here the periodic constant *a*=2*λ*_SP_/3, for which the *α* profile is continuous enough to generate SPP profiles (The quality of SPP profiles can be improved by using smaller periodic constant *a*, as discussed in Supplementary Fig. 4. The simulated and experimental SPP profiles for LCP/RCP incidence are shown in Fig. 2c–f, which faithfully realize the local SPP profiles within the target region, and indicate the two orbitals (LCP and RCP incidence) can be arbitrarily designed and are not necessarily bounded to beam displacement splitting comparable to a wavelength in real space or bounded to opposite k-space splitting relation between the two spins^18^,^32^,^33^,^42^.

Apart from the simple focusing, our current scheme can actually be used to construct far more complicated SPP profiles. In this example, we generate a triangle for LCP incidence and a cross shape for RCP incidence. A more complex SPP profile requires more nano-slots in storing the additional information (see Supplementary Note 4 and Supplementary Figs 5 and 6 for additional results on pattern complexity). We have employed more nano-slots (number of nano-slots for the current sample is 1.5 times of that of the last sample) with a larger ring with radii from 10 *λ*_SP_ to 13.3 *λ*_SP_ (Fig. 2h). Figure 2i–l present the simulated and experimentally achieved SPP profiles, and both clearly show the targeted triangle and cross SPP profile with LCP and RCP incidence (we note here that the target SPP profile for either LCP or RCP incidence is an ensemble of dipole-like focus spots arranged in the pattern of ‘triangle' or ‘cross', schematically shown in Fig. 2g.). To quantify the quality of the realized SPP profiles, we calculate the root-mean-square-deviation (RMSD) and also the Pearson product-moment (PPM) correlation between the experimentally observed and the theoretical SPP patterns (see Supplementary Note 5 and Supplementary Figs 7 and 8 for details). For ‘triangle' and ‘cross' pattern, RMSD are 0.18 and 0.17, or PPM coefficient of 0.50 and 0.40 (or 0.5 on average for all patterns in this work, see Supplementary Table 1). These measured merits of SPP patterns indicate our geometrical phase matching scheme is flexible enough for us to make complicated and independent profiles for the two spins, as an indication towards completely harnessing the plasmonic spin-Hall effect. The independent profiles can be arbitrarily complicated as long as they are local SPP orbitals, which can exist in the slot-free region. They are still subject to diffraction limit, however.

### Coherent control of SPP orbitals

As a direct implication of this complete control of spin splitting, these SPP profiles excited by the two circular polarizations can work coherently with each other by controlling the relative phase between the two polarizations. Here we demonstrate how to tune the amplitude of an orbital and to make ‘motion pictures' played by polarization rotation as illustrations of coherent control.

**Figure 3:**
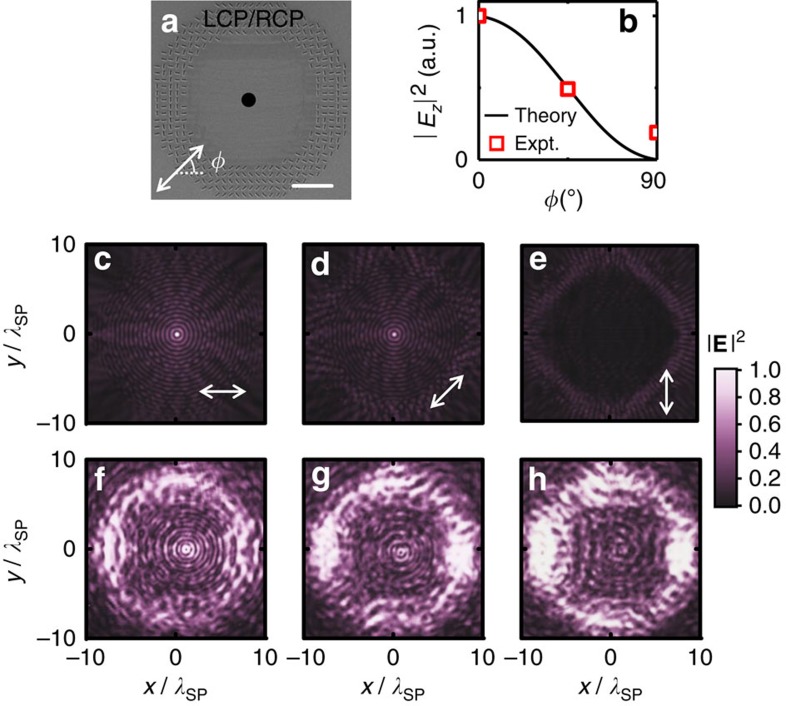
Coherent control of amplitude of a single orbital by varying *φ*. (**a**) Target SPPs for LCP and RCP orbitals both focus at (0,0). Scale bars, 4 μm in **a**. (**b**) Predicted (curve) 
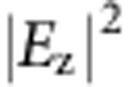
 and measured intensity of SPP profiles (symbols) at focus spot with varying *φ*. (**c**–**h**) are the simulated 
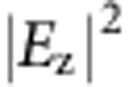
 and measured intensity profiles with varying *φ* (white arrows). Here we use same colour scale for SPP patterns with different incident polarization angles.

As a simple example of coherent control, we demonstrate how to tune the amplitude of an orbital by varying the linear polarization angle *φ* of the incident light. Here the orbitals for both spins are set to same one: a focal spot with 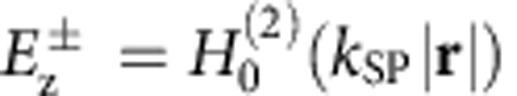. The generated SPP profile becomes 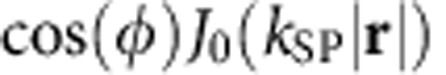 with linear polarization 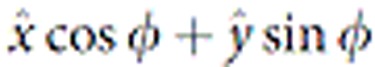. Again, we use the modified scheme to design and fabricate the sample shown in Fig. 3a. Full wave simulations and experiments are performed with varying *φ* in Fig. 3c–h. The measured intensity of the orbital (integrated around the focus spot) gradually varies from maximum to zero by tuning *φ* from 0° to 90°, with theoretical prediction cos^2^(*φ*) shown in Fig. 3b. In fact, we can further tune the maximum intensity to occur at a polarization-selective angle *ξ*, by multiplying 
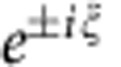
 on the LCP and RCP target profiles. The generated SPP standing wave in the target region becomes 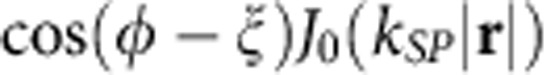. This amplitude control (with *ξ*) constitutes a single ‘pixel' for further construction.

**Figure 4:**
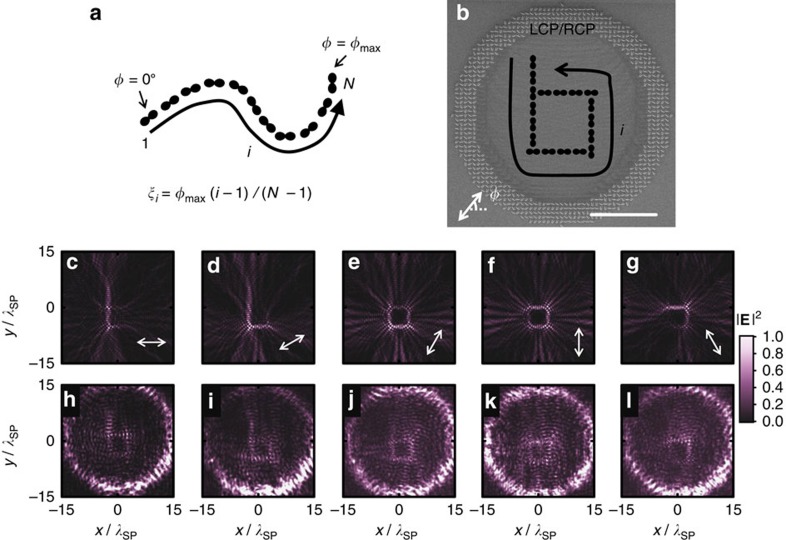
Motion pictures played by polarization rotation angle. (**a**) The scheme of Motion pictures, which is consisted by N pixels. Polarization-selective angle *ξ*_*i*_ increases along the path of pixels. (**b**) SEM image of fabricated sample of moving letter ‘b', (with *φ*_max_=120° and ring radii from 13.3*λ*_SP_ to 16.7*λ*_SP_). Scale bars, 10 μm in **b**. (**c**–**l**) Are the simulated 
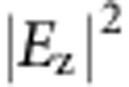
 and measured intensity profiles with varying *φ* from 0° to 120° (white arrows). The colour scales of SPP patterns with different incident polarization angle are the same.

**Figure 5:**
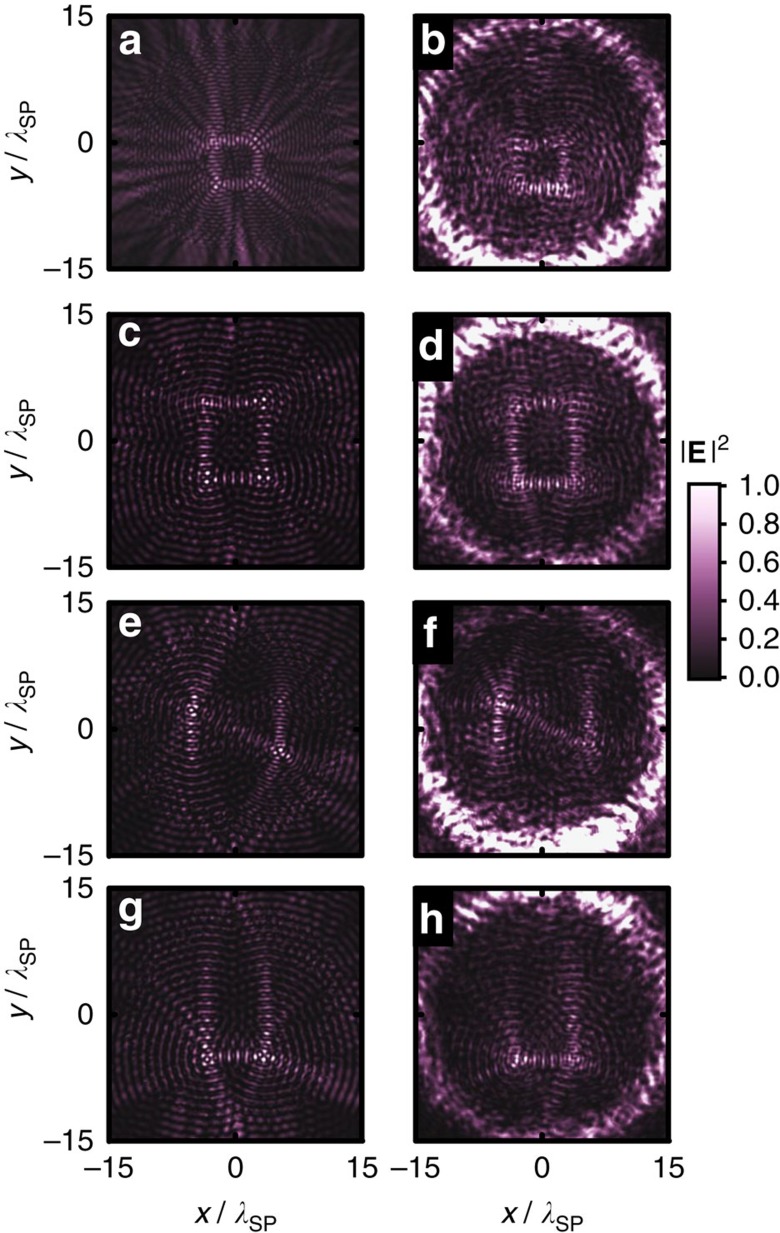
The static pictures of motion pictures. (**a**,**b**) letter ‘b', (**c**,**d**) letter ‘O', (**e**,**f**) letter ‘N', and (**g**,**h**), letter ‘U' with RCP incidence are the simulated 
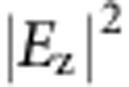
 profile/the measured intensity profile. The measured performance merit RMSD (PPM) are 0.15 (0.42) for letter ‘b', 0.17 (0.53) for letter ‘O', 0.16 (0.56) for letter ‘N' and 0.13 (0.53) for letter ‘U', respectively.

Such coherent control of orbitals including phase and amplitude can further allow us to design individual picture frames, which will only light up at different and designated polarization angle *φ*. By using geometric phases from the same set of atoms, we can construct motion pictures by tuning the incident polarization. Here we take an array of these pixels for illustration, and instruct the light to write an alphabet when the linear polarization angle *φ* is rotated. We set the target SPP profile as


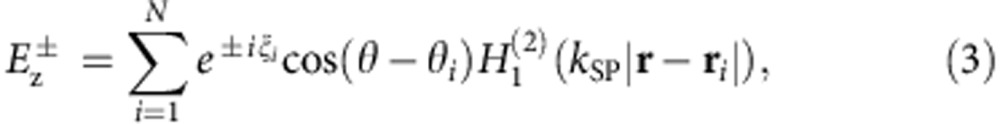


where *ξ*_*i*_=*φ*_max_(*i*−1)/(*N*−1) linearly increases along the path of pixels with index *i*=1 to *N* (Fig. 4a). It lights up the pixels in sequence with an example of letter ‘b' (Fig. 4b). Each pixel is a dipole-like focal spot with its direction *θ*_*i*_ aligned with the stroke joining successive pixels. To illustrate the dynamic behaviour, we plot the ‘time' frames of the simulated and measured SPP profiles with different values of *φ*. As *φ* increases, the profiles have their maximum intensity tracing the letter ‘b' in the counter clock-wise direction as designed (Fig. 4c–l). On the other hand, a ‘static' picture of the letter ‘b' is revealed by a RCP incidence (Fig. 5a,b). The polarization-selectivity at each pixel is lost in such a case. The target orbitals for both spins have the same amplitude (static picture) designed from equation (3) while the subtle difference in phases at the pixels, storing the time sequence information, is a complicated version of coherent intensity control between the static pictures from the two spins. Apart from a letter ‘b', a letter ‘O', a letter ‘N' and a letter ‘U' are also designed and fabricated for further illustrations, the simulated and measured static pictures of which are presented in Fig. 5c–h for RCP incidence. The measured RMSD are all below 0.2 with correlation coefficient around 0.5, which show a clear correlation with the designed SPP pattern. Their motion pictures are shown in Supplementary Figs 9–11. Both static and motion pictures faithfully realize the designed sequence in writing the letters.

## Discussion

In the present work, we have established a generic geometric phase scheme for SPP generation with geometric phase elements on a metasurface. This scheme provides us the freedom to independently control both the amplitude and phase of local SPP orbitals by two opposite spins. With this freedom, we have demonstrated arbitrary plasmonic spin-Hall effect not only about splitting of SPP orbitals but also about generating different controllable shapes of SPP profiles. We note that such arbitrariness in SPP orbital control (independence for two incident spins, with arbitrary amplitude and phase control at the same time) is provided by the spin nature of CP light together with our the geometric phase matching scheme (equations (1, 2, 3)). The usage of CP light allows us to avoid the so called ‘amplitude spatial-dispersion' problem of nano-slot arrays that the transmitted amplitude and phase though such nano-slots cannot be independently controlled. Although the V-shape and C-shape antennas^22^,^23^,^27^, can resolve such locked amplitude and phase problem with linear polarized incident light, the generated profiles for the two linear polarization in this case will then be unavoidably dependent on each other so that we lost the independence of the generated patterns between the two orthogonal polarizations.

On the basis of such flexible control of orbital shapes, we have further demonstrated how to make SPP orbitals with opposite spins work coherently to manipulate the amplitude of SPP orbitals, and allow us to design a ‘time' series of frames to make motion pictures by continuously varying the linear polarization angle of the incident plane wave. We note that apart from demonstrating continuous motion of an object (a spot in our case), we can also insert very different pictures between the initial and the final frame, so that each pattern only lights up at its particular polarization angle as shown in Supplementary Fig. 12. Such a flexible control is the direct consequence of that phase as well as amplitude of the individual orbitals can be manipulated. From the experimental results, there exists some noise in the measured SPP profiles indicating a lower performance than the simulated result. It mainly comes from two origins: the roughness of metal film, which causes more undesired SPP scattering; and the directly transmitted light from the nano-holes that can also lower the contrast. These can be further improved by better metal film deposition technique in the future works, such as Molecular Beam Epitaxy or single crystal growth technique. On the other hand, we can also fabricate larger rings of nano-particles to minimize the direct transmission from nano-slots to be detected (see Supplementary Fig. 13). Moreover, the effectiveness in realizing a designed SPP profile requires the data capacity stored by the nano-slots to be larger than the data capacity required by the SPP profile. Generally, the quality of the realized SPP profile depends on the ratio between the number of nano-slots and the number of focal spots in forming the SPP profile. Higher this ratio has a higher quality (PPM). The above capability of spin-enabled coherent control of SPP orbitals opens a unique and arbitrary way in polarization tunability of localized SPP, including controllable phase, intensity, and position of the SPP orbitals, and even making motion pictures. This technique may be exploited to further harness the SPP in near-field applications. For example, hot spots with tunable positions can be used as tip-free near-field scanning optical microscopy^47^, or plasmonic tweezers^48^ to trap and move micron size particles. Furthermore, we can use more tunable orbitals as coherent inputs of SPP logic devices (coherent logic gate, transistor, etc.) situated in the same area^49^, that is, the input coherence required on a microscopic SPP device is now translated to a required coherence of the LCP and RCP incident light (at the macroscopic scale), which can be realized easily.

## Methods

### Fabrication and experiemental set-up

A silver/MgF_2_/silver sandwich structure was fabricated for the experiment, as shown in Fig. 1d. A LiNbO_3_ substrate was successively sputtering-deposited with a 65-nm-thick silver layer, a 55-nm-thick MgF_2_ layer and a 45-nm-thick silver layer. The metasurface pattern was then drilled across the top silver film using a focused ion beam (FEI Strata FIB 201, 30 keV, 11 pA). A continuous semiconductor laser was used to excite the SPP on the metasurface. Here the bottom silver layer is used to block the direct transmission signal from the incident laser. The leaky radiation signal of SPP is collected through an oil-immersed micro-objective lens below the LiNbO_3_ substrate. The SPP propagation is imaged with a high resolution CCD.

### Equivalent 2D simulation

The numerical results in the present paper are obtained by simulating the *E*_z_ field in an equivalent 2D transverse electric (TE) wave simulation. The point dipole sources are replaced by equivalent 
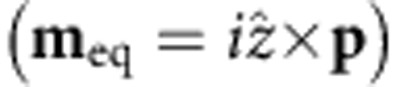
 magnetic dipole line source, since the 2D *E*_z_ SPP profile excited by a point source in three-dimensional is equivalent to dipolar line sources in 2D problem (see details in Supplementary Note 6). As long as the nano-slots can be treated as dipolar sources, this approximation is valid.

### Design scheme for complex pattern

In the present paper, all target SPP profiles are designed by an ensemble of focus spots, which can be generally expressed as 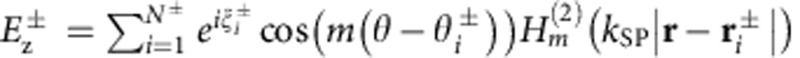, where *m* is the order of Hankel function and 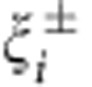, 
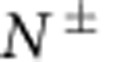
, 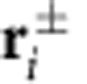, 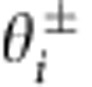 are the polarization-selection phases, total number of focus spots, locations and stroke direction of *i*-th focus spot with LCP/RCP incidence, respectively.

## Additional information

**How to cite this article:** Xiao, S. *et al*. Flexible coherent control of plasmonic spin-Hall effect. *Nat. Commun*. 6:8360 doi: 10.1038/ncomms9360 (2015).

## Supplementary Material

Supplementary Information Supplementary Figures 1-13, Supplementary Table 1, Supplementary Notes 1-6 and Supplementary Reference
