# Supplementary material for: Flexible coherent control of plasmonic spin-Hall effect
Source: Nat Commun. 2015 Sep 29;6:8360. doi: 10.1038/ncomms9360 (PMC4598558; doi:10.1038/ncomms9360)
Supplement: Supplementary Information — Supplementary Figures 1-13, Supplementary Table 1, Supplementary Notes 1-6 and Supplementary Reference [file ncomms9360-s1.pdf]

Supplementary Figures

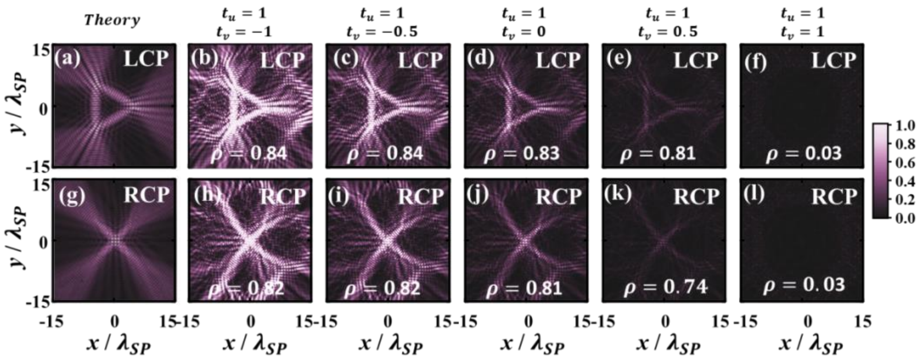

Supplementary Figure 1 | Theoretical and simulated $|E_z|^2$ profiles with varying t_u and t_v. (a-f) LCP incident wave and (g-l) RCP incident wave.

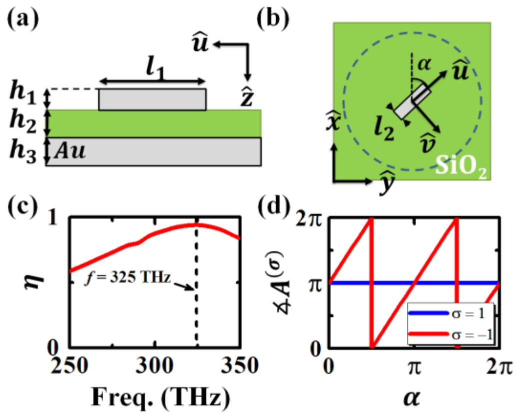

Supplementary Figure 2 | Designed nanoparticle with cross-CP conversion. (a) and (b): side and top view of the plasmonic particle on metal, with geometrical parameters defined as $h_1 = 50$ nm, $h_2 = 50$ nm, $h_3 = 100$ nm, $l_1 = 270$ nm, $l_2 = 110$ nm. The metal (gray color) is gold, and the dielectric spacer (green color) is SiO_2. (c) Cross-polarization conversion efficiency versus frequency, and (d) Geometric phase $arg\left(A^{(\sigma)}\right)$ versus orientation angle α with normal LCP incident light.

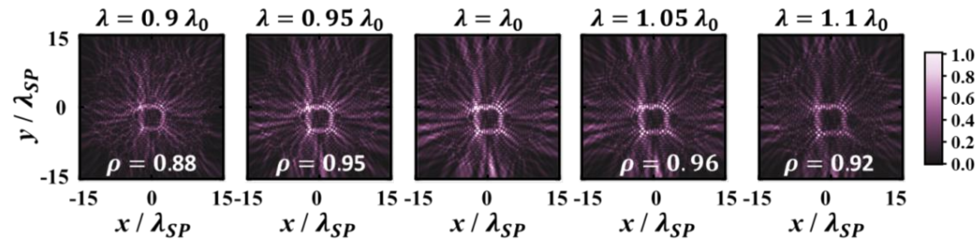

Supplementary Figure 3 | The wavelength dependency of the SPP pattern. SPP generated by a definite ring-shaped nano-slots array (with writing Letter "b"), where λ_0 is the working wavelength. We shift the wavelength from 0.9 λ_0 to 1.1 λ_0, where λ_0 is the working wavelength. Here, we can find when the wavelength is within 0.95 λ_0 to 1.05 λ_0, the SPP profiles are still realized very well and the performance merit ρ is still over 0.9, so that the fractional wavelength bandwidth of designed metasurface is about 0.1 λ_0.

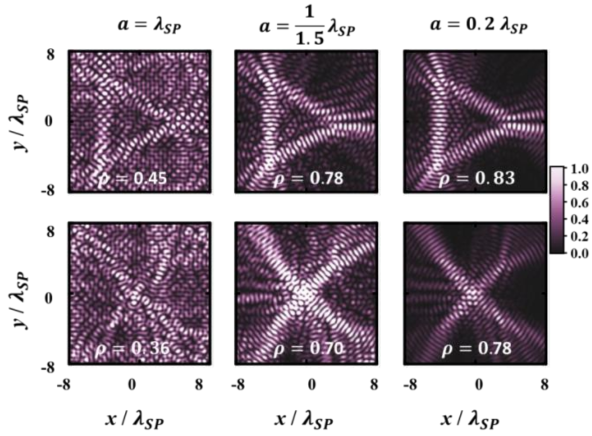

Supplementary Figure 4 | Effect of periodic constant on SPP qualities. The simulated SPP profiles for different atom density from $a = \lambda_{\mathrm{SP}}$ to $a = 0.2\,\lambda_{\mathrm{SP}}$ are presented here with LCP (upper panel) and RCP (lower panel) incident waves. We can find that the quality of SPP profiles can be improved by using a smaller periodic constant a. By increasing the atom density (from $a = \lambda_{\mathrm{SP}}$ to $a \ll \lambda_{\mathrm{SP}}$), the image quality as well as the performance merit can be enhanced. It also shows the trend when we decrease the lattice constant a from λ_{SP} to less than 0.2 λ_{SP}, which converges to the continuous limit. With more atoms, the patterns ("triangle" for LCP incidence in up panel and "cross" at RCP incidence in low panel) looks clearer with quantitative merit of roughly a two-fold improvement.Two reasons contribute to this behavior: (1) The more atoms in per $\lambda_{\mathrm{SP}} \times \lambda_{\mathrm{SP}}$ region means the more information can be provided so that the more accurate the SPP profile can be generated; (2) The larger atom density generally means the interference of co-CP term is not constructive.

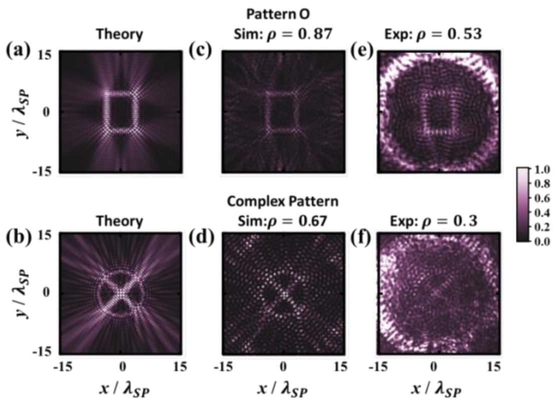

Supplementary Figure 5 | Effect of pattern complexity on SPP qualities. The theory ((a) and (b)), simulated ((c) and (d)), and experimental ((e) and (f)) SPP profiles for Pattern Letter O (simple pattern, up panel) and Complex Pattern (low panel) with CP incident wave.

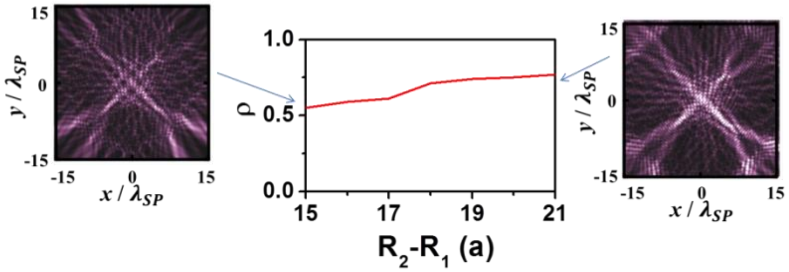

Supplementary Figure 6 | Effect of number of atoms on SPP. The simulated PPM (ρ) as function R_1 for a complex SPP pattern (“cross” for both LCP and RCP).

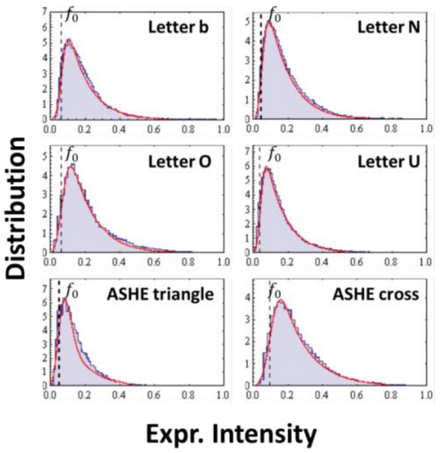

Supplementary Figure 7 | Experimental intensity distributions. The intensity distributions of experiments (blue curves), analytical (theory plus a background noise with mean f_0 and standard deviation σ_n, red curves), and dashed lines denote the background field intensity f_0.

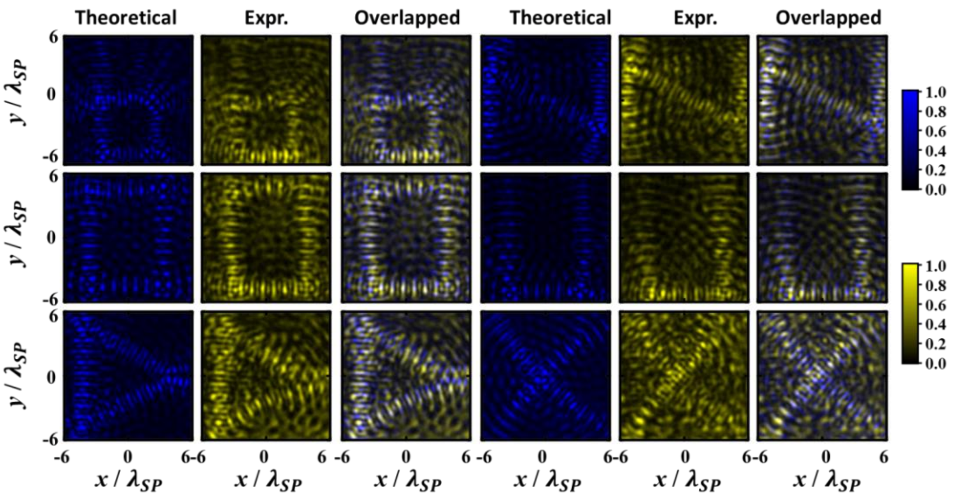

Supplementary Figure 8 | Comparing theoretical and experimental patterns. The SPP fringes of design (blue) and the fringes of experiment (yellow) agree in position and in the intensity in white color in the overlapped images.

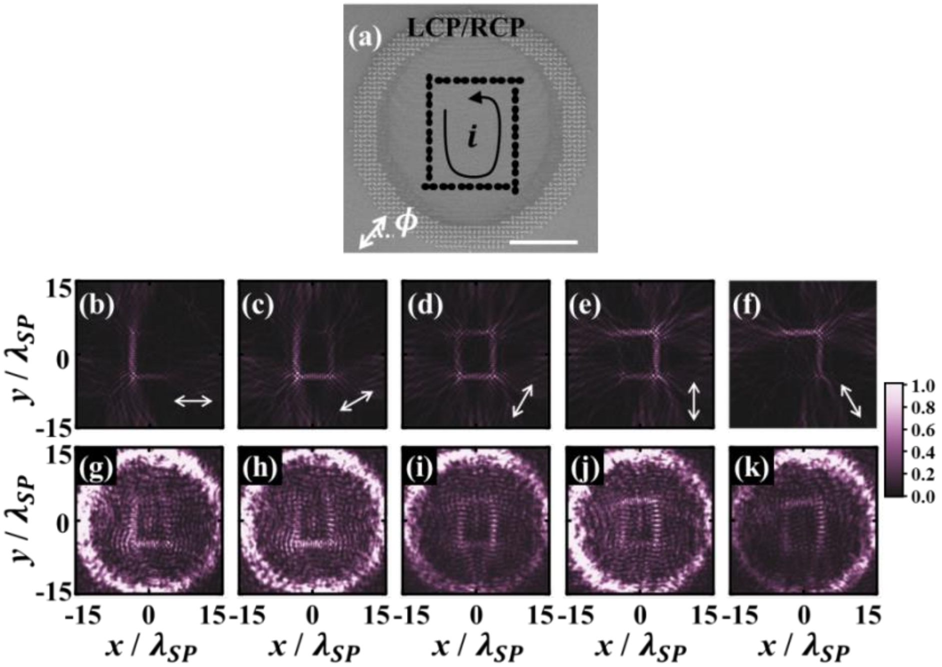

Supplementary Figure 9 | Moving picture of letter "O". (a) SEM image of fabricated sample, (b-f) simulated $|E_z|^2$ and (g-k) measured intensity of SPP profile being played by increasing the polarization rotation angle ϕ. Scale bars, 10 μm in **a**.

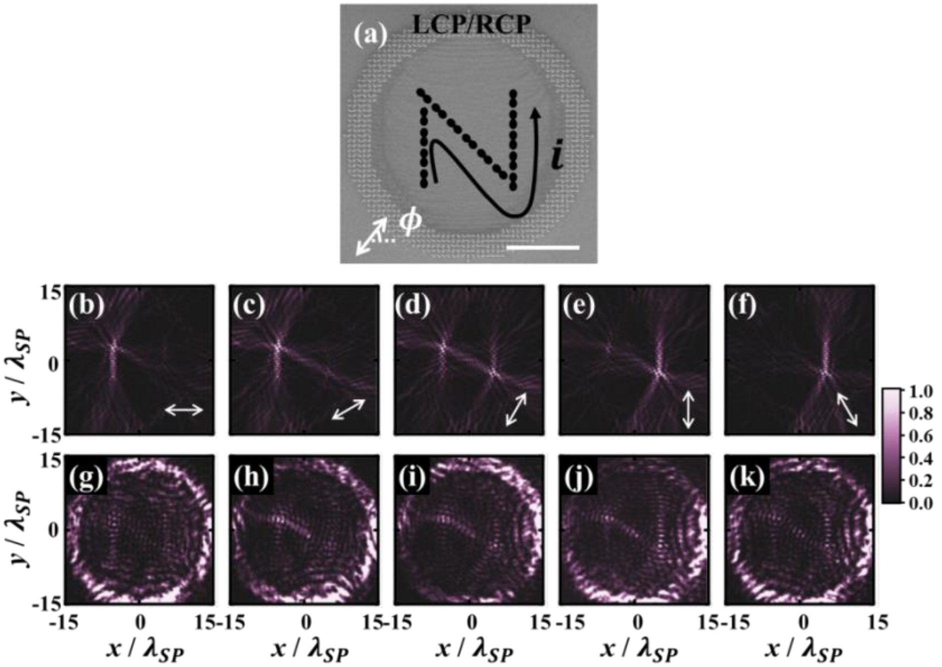

Supplementary Figure 10 | Moving picture of letter "N". (a) SEM image of fabricated sample, (b-f) simulated $|E_z|^2$ and (g-k) measured intensity of SPP profile being played by polarization rotation angle ϕ. Scale bars, 10 μm in **a**.

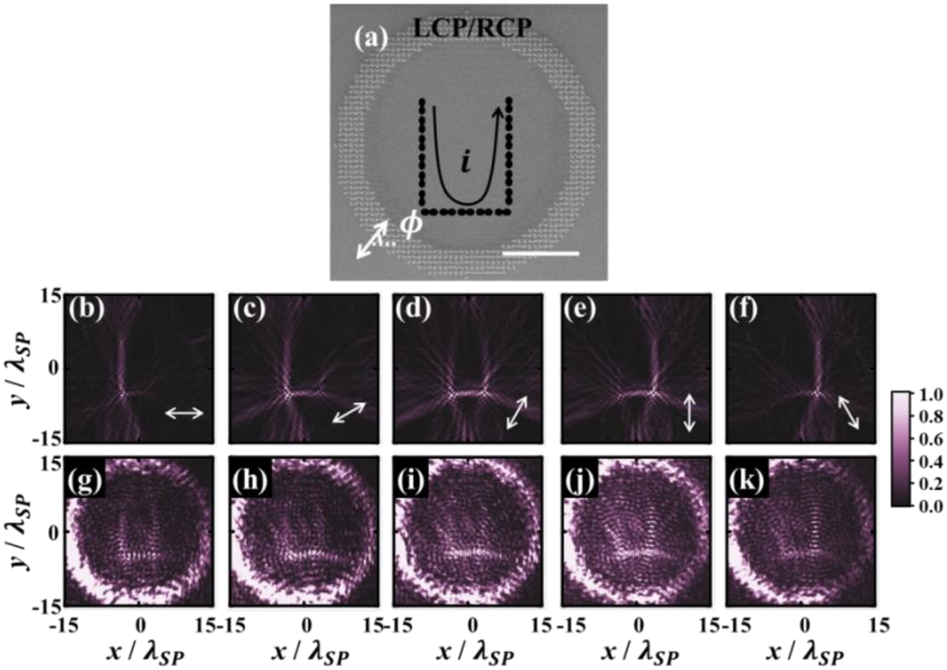

Supplementary Figure 11 | Moving picture of letter "U". (a) SEM image of fabricated sample, (b-f) simulated $|E_z|^2$ and (g-k) measured intensity of SPP profile being played by polarization rotation angle ϕ. Scale bars, 10 μm in **a**.

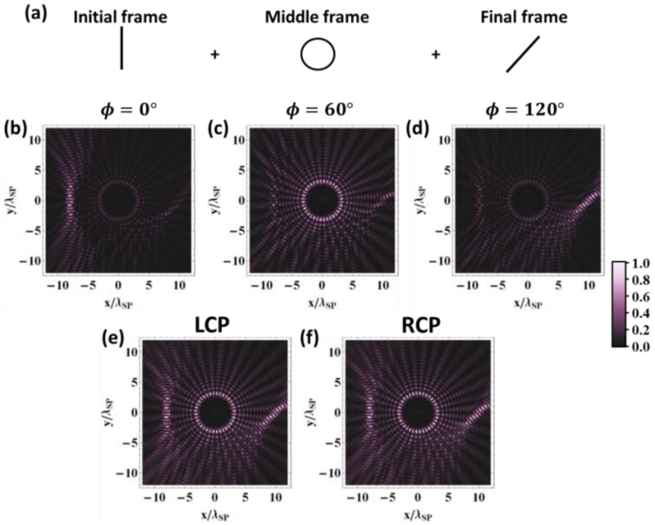

Supplementary Figure 12 | Motion picture with discrete frames. We can insert a totally different pattern (a circle in the middle with $\xi = 60°$) between the initial frame (a bar on the left with $\xi = 0°$) and the final frame (a bar on the right with $\xi = 120°$). Each pattern only lights up at when particular polarization angle equals to its ξ. (a) Initial, middle, and final frames with different patterns. (b)-(c) are $|\mathbf{E}|^2$ filed profiles with LP incident wave as function of polarization angle ϕ (b) 0°, (c) 60°, (d) 120°. The color scales are the same for different polarization angles. (e) and (f) are $|\mathbf{E}|^2$ intensity profiles for LCP and RCP incidence.

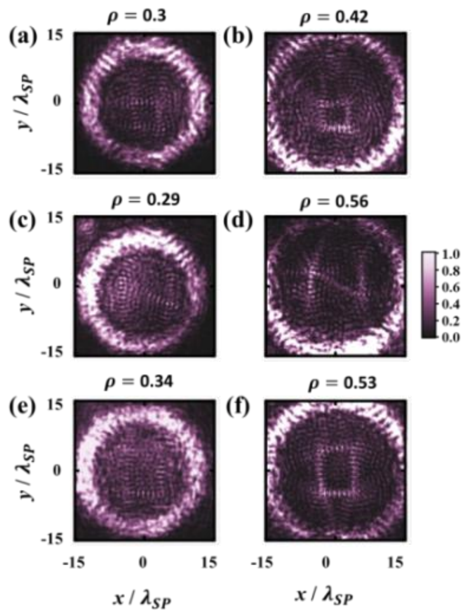

Supplementary Figure 13 | Improving the image quality by a larger rings of particles The measured SPP pattern and ρ of the orignal ring (left panel, $R_1 = 20\ a$) and a large ring of particles (right panel, $R_1 = 25\ a$) for letter b, N, O where R_1 is the inner radii of the ring of nanoparticles. The results show an enhancement of image quality, by increasing the size of the ring. We can clearly find that the noise (small spots not belonging to the target pattern) is reduced, also revealed by the merit ρ (correlation between experimental and target pattern) with roughly 50% enhancement.

Supplementary Tables

	Letter b	Letter N	Letter O	Letter U	ASHE triangle	ASHE cross
$\boldsymbol{f_0}$	0.061	0.045	0.063	0.039	0.067	0.096
$\boldsymbol{\sigma_\mathrm{n}}$	0.025	0.023	0.033	0.020	0.047	0.040
**Contrast**	0.88	0.91	0.88	0.92	0.88	0.82
**Contrast-to-noise ratio**	13.1 dB	16.1 dB	14.4 dB	16.9 dB	13.0 dB	13.6 dB
**RMSD**	0.15	0.16	0.17	0.13	0.18	0.17
$\boldsymbol{\rho}$	0.42	0.56	0.53	0.53	0.50	0.40

Supplementary Table 1 | Statistic study of experimental results and performance merits. Fitted parameters of the mean f_0, standard deviation σ_n of the background noise, measured contrast, contrast-to-noise ratio, measured RMSD between experimental and theoretical SPP profiles, and measured PPM correlation coefficient between theory and experimentally observed SPP profiles.

Supplementary Notes

Supplementary Note 1| Deviation of geometric phase matching rule.

We excite ring-shaped nano-slots array with normal incident light. Each of them oscillates as an electric dipole moment $\mathbf{p}$ (with harmonic factor $e^{-i\omega t}$) as

$$\mathbf{p} = \frac{1}{2}\Big((t_u + t_v)(\hat{\mathbf{x}} \pm i\hat{\mathbf{y}}) + (t_u - t_v)e^{\pm 2i\alpha}(\hat{\mathbf{x}} \mp i\hat{\mathbf{y}})\Big), \quad (1)$$

where t_u/t_v denotes the transfer amplitude in $\hat{\mathbf{u}}/\hat{\mathbf{v}}$ axis from a plane wave if it is linearly polarized along a principal axis $\hat{\mathbf{u}}/\hat{\mathbf{v}}$ of the particle with orientation angle α. Upper/lower sign denotes LCP/RCP ($\hat{\mathbf{x}} + i\hat{\mathbf{y}}/\hat{\mathbf{x}} - i\hat{\mathbf{y}}$) incidence and the same convention will be used in the following. The second (cross-polarization) term carrying a geometric phase $\pm 2\alpha$ allows us to generate a designated SPP profiles. Next, we would like to ensure constructive interference for the radiations from the different dipole moments by requiring

$$arg(\mathbf{p}^* \cdot \mathbf{E}_{\mathrm{T}}) = \text{constant}, \quad (2)$$

where $\mathbf{E}_{\mathrm{T}}$ is the transverse electric field of the SPP and is proportional to $\nabla_{\mathrm{T}} E_z$ (see Supplementary Eq. 3-8 for the derivation). The vector nature of Supplementary Eq. (2) is necessary as the SPP can have different propagation direction on the surface. Here, we only use the cross-polarization term in Supplementary Eq. (1) to construct SPP profile. The local field generated by the co-polarization term will not construct meaningful patterns, as it does not carry Berry phase [See Supplementary Note 3 for the numerical evidence]. By putting the cross-polarization term of Supplementary Eq. (1) into Supplementary Eq. (2), we obtain the geometric phase matching rule (or the function f) in Eq. (1) in text to design the orientation (α) profile of the plasmonic atoms.

Supplementary Note 2| Designing recipe for nano-slots array.

Designing recipe involves the following steps:

(1) The target standing wave patterns within an interested region can be decomposed into a part radiating into the region and a part radiating away from the region. We label the part radiating into the region as $E_{\mathrm{z}}^{\pm}$. The superscript indicates the pattern for the particular incident spin.

(2) Superimpose the two target patterns by $E_z = E_z^+ + (E_z^-)^*$, which is used as the input to the geometric phase matching rule with LCP incidence: $\alpha(x,y) = f_+(E_z) = \frac{1}{2} arg\left(\partial_x E_z + i\partial_y E_z\right)$. It gives the orientation angle of a plasmonic atom at the position (x,y). Similarly, for RCP incidence, we can use $E_z = E_z^- + (E_z^+)^*$ as input to geometric phase matching rule with RCP incidence and it just gives the same answer on the orientation profile.

Here, we would like to take the arbitrary and spin-dependent SPP profiles (Fig. 2a-f in text) as a example. We want LCP incident wave to generate two focal spots at $\mathbf{r_1} = (1,-2)\lambda_{\mathrm{SP}}$ and $\mathbf{r}_2 = (-2,0)\lambda_{\mathrm{SP}}$, and RCP incident wave to generate single focal spots at $\mathbf{r}_3 = (0,2)\lambda_{\mathrm{SP}}$, so that the generated SPP function should be $J_0(k_{\mathrm{SP}}|\mathbf{r}-\mathbf{r_1}|) + J_0(k_{\mathrm{SP}}|\mathbf{r}-\mathbf{r_2}|)$ for LCP and $J_0(k_{\mathrm{SP}}|\mathbf{r}-\mathbf{r_3}|)$ for RCP incidence. Then, by taking the part radiating into the region as the target waves, and we obtained $E_z^+ = H_0^{(2)}(k_{\mathrm{SP}}|\mathbf{r}-\mathbf{r_1}|) + H_0^{(2)}(k_{\mathrm{SP}}|\mathbf{r}-\mathbf{r}_2|)$, and $E_z^- = H_0^{(2)}(k_{\mathrm{SP}}|\mathbf{r}-\mathbf{r_3}|)$. Finally, we insert $E_z^+ + (E_z^-)^*$ into Eq. (2) in text to obtain the α profile.

Supplementary Note 3 | The simulated SPP profile with varying transfer amplitude t_u, t_v.

In this section, we will present the simulated SPP with varied t_u and t_v for grids of nano-slots used in this paper, to demonstrate the co-polarization term in Supplementary Eq. (1) is not significant in generating SPP profiles in this work. Here, we take an the example of instructing LCP to generate a “triangle” and RCP to generate a “cross” pattern as in Supplementary Fig. 1. From $t_u = 1$, $t_v = -1$ (atom with complete cross-polarization conversion) to $t_u = 1$, $t_v = 0$ (intermediate case: the case in the paper, just acting like a polarizer) to $t_u = 1$, $t_v = 1$ (the worst case with complete co-polarization conversion). We find the SPP profile fit the target SPP very well until nearly approaching the case of $t_u = 1$, $t_v = 1$ (with complete co-polarization conversion and no geometric phase). These simulation results indicate the field intensity of the SPP profile generated by the co-polarization term is much weaker than the one generated by the cross-polarization term due to the absence of constructive interference for the field carried by the co-polarization term. In addition, we also calculate the performance merit, correlation

merit ρ (See the definition in Supplementary Note 5), for each case and have found that the performance merit remains at a high value even for $t_u = 1, t_v = 0.5$ (90% of total power of SPP is carried by the co-polarization instead of the cross-polarization term). These results confirmed that the radiation pattern from co-polarization term can be neglected for nano-slots used in this work.

Although we have limitation in current fabrication facilities, we note that the coupling can be further enhanced in future designs by using plasmonic particles with higher conversion efficiencies to cross-polarization term [See Supplementary Fig. 2]. Supplementary Fig. 2 (a) and (b) show a typical structure which radiates nearly only the cross-polarization term at a particular working frequency. It consists of a gold bar, sitting on a gold surface with a quartz spacer layer (green color, with dielectric constant 2.1 and detailed structural parameters in the caption of Supplementary Fig. 2). The topological structure of the SPP excited by a normally incident plane wave can be visualized by integrating the SPP field profile at a fixed radius $R = 810\text{nm}$ (dashed circle in Supplementary Fig. 2(b)) using $A^{(\sigma)} = \int e^{-i\sigma\phi} E_z(R, \phi) d\phi$. Then, the cross-polarization conversion efficiency is defined by $\eta = |A^{(\mp 1)}|^2 / \sum_{\sigma=-1}^{1} |A^{(\sigma)}|^2$ for both LCP and RCP incident light. This efficiency is extracted through full-wave simulations (COMSOL Multiphysics) and is plotted in Supplementary Fig. 2(c). It exhibits a broadband peak with the highest conversion efficiency of around 93% at 325 THz, the optimal working frequency to make the co-polarization term negligible. By rotating the plasmonic particle by an angle α in the simulation, the generated SPP phase versus α is plotted in Supplementary Fig. 2(d) with $arg(A^{(1)}) \cong 0$ and $arg(A^{(-1)}) \cong 2\alpha$, which further validates the geometric phase (due to particle orientation) of the cross-polarization term.

Supplementary Note 4| Effect of pattern complexity on the quality of generated SPP profiles.

As we show in Supplementary Fig. 5, the simulated SPP profile of simple pattern (pattern O) in Supplementary Fig. 5 (a) is more clear than the SPP profile of complicated pattern in Supplementary Fig. 5(c). The simulated (measured) performance merit of simple pattern is 0.87 (0.53), and merit of complex pattern is only 0.67 (0.3), where the performance merits are defined in Supplementary Note 5.

The image quality of complex pattern can be enhanced by employing more atoms to provide more "information". As shown in following Supplementary Fig. 6, the correlation coefficient ρ increase from 0.55 to 0.77 when the inner radius of the particle ring R_1 with the ring thickness fixed as $5a$. From the perspecitive of data capacity, we need the data capacity required by the SPP profile design, which increases with the number of spots in forming the design, to be smaller than the data capacity provided by the nano-slots, which is increased by the number of nano-slots (plasmonic particles) here.

Supplementary Note 5| Definition of performance merits and contrast.

Measuring Overall Image Quality

The deviation between an experimentally realized SPP profile and the calculated (simulation) profile is introduced at both the fabrication and experimental stages, which can be shown by plotting the statistics of the pixel intensities as a whole before the correlation between the two profiles are investigated using Root-Mean-Square-Deviation (RMSD) and the Pearson-product-moment (PPM) coefficients. Supplementary Fig. 7 shows the pixel intensity distributions for the 6 patterns experimentally demonstrated in the main text (the blue area). While the decay "tail" at higher intensities comes from the statistics of the simulation, the rising edge with a finite slope can be treated as a background noise being added to the simulation. With such an empirical model, we can fit the noise part, being assumed as a normal distribution of mean f_0 and standard deviation σ_n, with the calculated intensity distribution from simulation. The fitted results are shown as the "analytical" curves in red color in the same figure while the fitted parameters of the background noise are shown in Supplementary Table 1.

The intensity distributions and their fitted parameters immediately allow us to quantitatively investigate the overall image qualities of the various SPP profiles plotted in the main text. In this work, as f_0 is actually roughly the background intensity observed inside the ring of nano-slots, we choose a contrast-measure defined by $(I_\mathrm{max} - f_0)/(I_\mathrm{max} + f_0)$ where I_max is the maximum intensity of the measured signal (the value "1" in the distributions). It can range from 0 to 1 (the ideal maximum contrast without background noise). Moreover, the contrast-to-noise ratio is defined by $(I_\mathrm{max} - f_0)/\sigma_\mathrm{n}$. They are listed in Supplementary Table 1 for the various measured SPP profiles. The

observed contrasts are all above 0.8, indicating the background noise intensity is already very small. We note that a small background intensity is unavoidable in a practical experiment. It can come from ambient light, a small fraction of transmitted/scattered light rather than the SPP, or electrical noise.

Measuring Deviation of SPP profiles

Here, we employ Root-Mean-Square-Deviation (RMSD) and the Pearson product-moment (PPM) coefficient to measure the correlation between the calculated and the experimental SPP profiles. RMSD is calculated by $\sqrt{E[(X-S)^2]}$ between experimental data X and calculated data S directly, within a region of $12\lambda_{\mathrm{SP}} \times 12\lambda_{\mathrm{SP}}$ just inside the ring of nano-slots. The RMSD for the various SPP profiles are shown in Supplementary Table 1.

On the other hand, the PPM correlation coefficient is defined by $\rho = E[(S-\mu_S)(X-\mu_X)]/(\sigma_S\sigma_X)$ with μ and σ being the mean and standard deviation of the experimental (X) and theoretical (S) data. Such figure-of-merit $\rho = 1$ indicates a perfect correlation, and 0 indicates no linear relation between two variables. The merit for the various SPP patterns are listed in Supplementary Table 1, showing a clear correlation between the experimental and theory patterns. Furthermore, we plot the associated theory (blue color), experimental (yellow color) and their overlapped patterns in achieving this level of ρ in Supplementary Fig. 8. The results show that the theoretical patterns and the experimental patterns are matched not only for the general shapes but also for almost all fringes of the SPP profiles [the positions with both blue and yellow colors overlapped as white color for similar intensities]. We note that the agreement of these microscopic features (the SPP fringes) is more challenging than (and beyond) the agreement of the general shapes since the detailed intensities of the fringes are sensitive to the experimental and fabrication situation. The details of the fringes are already near the resolution limit of our optical system as well. As a further note, the RMSD has a lower bound $\sigma_X\sqrt{2-2\rho}$, which only accounts for the correlation part but not the part of background noise. The difference between the observed RMSD and the bound is only about 0.03 for different samples on average. Such a small difference is due to the unavoidable background noise (the finite f_0 and σ_{n} in Supplementary Fig. 7) and also indicates the large contrast and the validity of using ρ as a figure of merit.

Supplementary Note 6| SPP wave equation and equivalent 2-D simulation.

We consider a stratified layer system with permittivity $\epsilon(z)$, which supports our interested surface mode, propagating on the x-y plane, with dispersion

$$k_x^2 + k_y^2 = k_{\mathrm{SP}}^2. \tag{3}$$

The local normal wave number $k_z(z)$, a function of z, is given by

$$k_z(z) = \sqrt{k_0^2\epsilon(z) - k_{\mathrm{SP}}^2}. \tag{4}$$

We also assume the surface mode has only in-plane magnetic field. Then the magnetic field in an infinitesimally thin layer of permittivity $\epsilon(0)$ at $z = 0$ can be generally expanded into

$$\mathbf{H}(x, y, z) = \mathbf{H}_{\mathrm{T}}(x, y)(\cos k_z(0)z + b \sin k_z(0)z), \tag{5}$$

where $\mathbf{H}_{\mathrm{T}} = \hat{\mathbf{x}}H_x + \hat{\boldsymbol{y}}H_y$ is the transverse magnetic field and b is a constant property of the SPP mode. By putting Supplementary Eq. (5) into the Maxwell's equations and assume all the fields with separation of variables between (x, y) and z, we obtain the expansion form of electric field

$$\mathbf{E}(x, y, z) = \mathbf{E}_{\mathrm{T}}(x, y)(\cos k_z(0)z - (1/b) \sin k_z(0)z)$$
$$+\hat{\mathbf{z}}E_z(x, y)(\cos k_z(0)z + b \sin k_z(0)z), \tag{6}$$

where $\mathbf{E}_{\mathrm{T}} = \hat{\mathbf{x}}E_x + \hat{\mathbf{y}}E_y$ is the transverse electric field while the Maxwell's equations (Heaviside-Lorentz units and time factor $e^{-i\omega t}$) can be expressed as a scalar wave equation

$$\nabla_{\mathrm{T}}^2 E_z(x, y) + k_{\mathrm{SP}}^2 E_z(x, y) = 0 \tag{7}$$

with

$$\mathbf{E}_{\mathrm{T}}(x, y) = \frac{ik_0\epsilon(0)}{k_{\mathrm{SP}}^2} Z(0)\nabla_{\mathrm{T}} E_z(x, y), \quad \mathbf{H}_{\mathrm{T}}(x, y) = \frac{1}{Z(0)} \hat{z} \times \mathbf{E}_{\mathrm{T}}(x, y) \tag{8}$$

and $Z(0) = \frac{bk_{\mathrm{z}}}{ik_0\epsilon(0)}$ is defined as the surface impedance at $z = 0$.

We would like to find the SPP profile excited by a plane wave normally incident on an array of nano-slots sitting on a metal plate. Each particle is assumed to be subwavelength in size and oscillate as a horizontal (in-plane) electric dipole moment $\mathbf{p} = p_x\hat{\mathbf{x}} + p_y\hat{\mathbf{y}}$. It acts as a point source to excite SPP on a metal surface with E_z-profile[1]

$$E_z = i\gamma H_1^{(1)}(k_{\mathrm{SP}}|x\hat{\boldsymbol{x}} + y\hat{\boldsymbol{y}}|)\mathbf{p} \cdot \hat{\boldsymbol{\rho}}, \tag{9}$$

where γ is the coupling factor and $H_n^{(1)}$ is the first kind of the Hankel function of order n. It has the same form for the E_z radiation from an equivalent magnetic dipolar line source (horizontal $\mathbf{m}_{\mathrm{eq}}$, invariant in z) in a medium of wavenumber k and intrinsic impedance η:

$$E_z^{(\mathrm{eq})} = \left(\hat{\boldsymbol{\rho}} \times \mathbf{m}_{\mathrm{eq}}\right)_z \frac{k_0^2 \eta}{4} H_1^{(1)}(k|x\hat{\boldsymbol{x}} + y\hat{\boldsymbol{y}}|), \tag{10}$$

Therefore, we can set

$$\mathbf{m}_{\mathrm{eq}} = i\hat{\mathbf{z}} \times \mathbf{p}. \tag{11}$$

The excited SPP field from the ensemble of the electric dipole moments (or the α profile) can then be simulated by putting the equivalent magnetic dipoles in a background homogeneous medium of wave number k_{SP}, satisfying Supplementary Eq. (4), within an in-plane TE wave (E_z) simulation (2D). The equivalent simulation will give the same E_z-profile radiated from the electric dipole moments up to a global scaling factor.

Supplementary Reference

1. Mueller, J. B., & Capasso, F. Asymmetric surface plasmon polariton emission by a dipole emitter near a metal surface. *Phys. Rev. B* **88,** 121410 (2013).
